# Vitamin A-first dose supplement coverage evaluation amongst children aged 12–23 months residing in slums of Delhi, India

**DOI:** 10.4103/0301-4738.53056

**Published:** 2009

**Authors:** Sandeep Sachdeva, Utsuk Datta

**Affiliations:** Ophthalmology division, Directorate General of Health Services, Ministry of Health and Family Welfare, Nirman Bhawan, New Delhi-110108, India; 1 Department of Education and Training, National Institute of Health and Family Welfare, Munirka, New Delhi-110067, India

**Keywords:** Immunization and evaluation, vitamin A

## Abstract

**Objective:**

To determine vitamin A-first dose supplement coverage in children aged 12–23 months and to find out its correlates with selected variables.

**Materials and Methods:**

The 30-cluster sampling technique based on probability proportional to size advocated by the World Health Organization was used to assess vitamin A-first dose supplement amongst 210 children in the age group of 12–23 months residing in slums of a randomly selected municipal zone of Delhi during October to November 2005.

**Results:**

Only 79 (37.6%) children out of 210 had received vitamin A-first dose supplement. Further analysis of 79 children was carried out with regard to selected variables like religion, gender, birth order, place of birth, immunization status and literacy of mother. These analyses showed that 71 (89.9%) were Hindu and eight (10.1%) were non-Hindu (*P* = 0.04). Nearly 44 (55.7%) males and 35 (44.3%) females had received vitamin A (*P* = 0.74). The proportion of children born in a health institution who received first dose (57%) of vitamin A supplementation was significantly higher than of those who were born at home (43%) (*P* < 0.001). Similarly, higher proportion of children with birth order-one (48.1%) in comparison to birth order-three or above (26.6%) received vitamin A (*P* < 0.001). Thirty children though fully immunized for vaccine-preventable disease up to the age-of-one year had not received vitamin A-first dose supplement, suggesting that an opportunity had been missed. The association between receipt of vitamin A by the child and literacy status of mother was found to be significant (*P* < 0.001).

**Conclusion:**

The study reflects low coverage of Vitamin A supplement.

Vitamin A is necessary not only for prevention of xerophthalmia but also for preserving integrity and maintaining the function of several organs in the body. Available evidence has established the role of vitamin A in preventing childhood morbidity and mortality.[1,2] Vitamin A deficiency is a major cause of morbidity and mortality in India and other developing countries.[3] An estimated 5.7% children in India suffer from eye signs of vitamin A deficiency.[4] Although, vitamin A deficiency can occur in any age group, the most serious effects are usually seen in the preschool children.[5] Vitamin A requirement in the fast-growing age group of two to four years is the greatest since dietary intake is precarious and illnesses such as diarrhea, acute respiratory tract infection and measles, which deplete vitamin A reserves, are common. Currently, vitamin A deficiency is considered to be a public health problem in selected geographical areas in India with superimposed wide variations within the regions.

Heartily, there is a scientific evidence of declining trends of vitamin A deficiency in the country.[6] Under vitamin A supplementation program that is integrated through Reproductive and Child Health (RCH) program and now National Rural Health Mission (NRHM), children between nine and 36 months of age are to be provided with vitamin A solution every six months starting with 100,000 IU at nine months of age with measles vaccination and subsequently 200,000 IU every six months till 36 months of age. With rapid urbanization in India and one of the highest growth rates in the world, around 27.8% of the population is forced to reside in urban slums (Census 2001). As the slums are considered to be high-risk areas in terms of healthcare delivery, an attempt was made to determine vitamin A-first dose coverage amongst children (12–23 months) residing in slums of Delhi and to explore its association with selected variables.

## Materials and Methods

The 30-cluster sampling technique based on probability proportional to size advocated by the World Health Organization (WHO) was used to assess coverage of vitamin A-first dose supplement.[7] Out of 12 municipal zones in Delhi, one was selected randomly i.e. south municipal zone. List of all the slum clusters existing in the south municipal zone was procured from the municipal office (Annexure I). The approximate population residing in these slum clusters was 4,29,130. A cluster sampling is a two-stage random sampling technique i.e. selection of cluster and identification of children in the selected cluster. Steps involved were listing of slum clusters along with their population; calculating cumulative population for each cluster; determining sampling interval; selecting a random number which was less than or equal to sampling interval; this represented the first cluster; by adding sampling interval to the selected random number, second and then subsequent clusters were selected. A total of 30 such clusters were chosen this way. After selection of a cluster, first household was selected randomly and then subsequent household using right hand approach. From each selected slum cluster, seven eligible children were covered thus making a total sample size of 210 (30 × 7). Resident children in the age group of 12–23 months who were born between the reference period of October 1, 2003 and September 30, 2004 were enumerated from each household. Based on the documentary evidence/recall of mother regarding vitamin A-first dose received by the eligible child, data was recorded in the pre-structured proforma (Annexure II). Selected information related to religion, sex, place of birth, birth order, immunization status and education of mother was also recorded. Data was collected during October-November 2005 by a single investigator and analyzed by calculating percentages and degree of association (chi-square test) using SPSS software.

## Results

Of the total eligible subjects contacted, only one refused to participate in the study. Hence, next eligible child was contacted in the same cluster. Out of 210 study subjects, there were 175 (83%) Hindu and the rest were non-Hindu. There were 120 (57.0%) male and 90 (43.0%) female children amongst study subjects. Nearly three-fifth (126) children were born at home with the rest (84) in health institutions. The birth order of children was one, two, three (or above) as 64 (30.4%), 63 (30.0%) and 83 (39.6%) respectively. There were nearly 50.0% children fully immunized for vaccine-preventable diseases up to the age-of-one year whereas 23% were never taken for immunization. There were 74 (35%) mothers who were literate.

It was observed that only 79 (37.6%) children out of 210 had received vitamin A-first dose supplement in the community Table 1. Further analysis of 79 children was carried out with regard to selected variables. This showed that 71 (89.9%) were Hindu and eight (10.1%) were non-Hindu (*P* = 0.04). Nearly 44 (55.7%) males and 35 (44.3%) females had received vitamin A (*P* = 0.74). The proportion of children born in health institutions who received first-dose (57%) of vitamin A supplementation was significantly higher than children born at home (43%) (*P* < 0.001). Similarly, higher proportion of children with birth order-one (48.1%) in comparison to birth order-three or above (26.6%) had received vitamin A (*P* < 0.001).

**Table 1:** Association of vitamin A-first dose coverage with selected variables

Variable	YES n=79 (%)	NO n=131 (%)	χ^2^ value	*P* value
Religion				
Hindu	71 (89.9)	104(59.4)	3.90	0.048
Non-Hindu	8(10.1)	27(77.1)		
Sex				
Male	44 (55.7)	76 (63.3)	0.10	0.742
Female	35 (44.3)	55(61.1)		
Place of birth				
Health institution	45 (57.0)	39 (46.4)	15.18	<0.001
Home	34 (43.0)	92 (73.0)		
Birth order				
1	38(48.1)	26(19.9)	19.20	<0.001
2	20 (25.2)	43 (32.8)		
3 or above	21 (26.6)	62 (47.3)		
Immunization status				
FI*	74 (93.7)	30 (22.9)	37.52	<0.001
PI** or NI***	5 (6.3)	101 (77.1)		
Education status of mother				
Literate	38(48.1)	36 (27.5)	9.18	0.002
Illiterate	41 (51.9)	95 (72.5)		

* FI (Fully immunized),

** PI (Partially Immunized),

*** NI (Non-immunized) column %

The child can receive vitamin A-first dose independent of immunization status, however, from the point of view of operational feasibility, a child is administered the first dose along with measles vaccine. It was noted that 30 children though fully immunized for vaccine-preventable disease up to age-one had not received vitamin A-first dose supplement. The proportion of children receiving vitamin A was slightly higher for illiterate mothers (51.9%) than literate mothers (48.1%). However, overall relationship of literacy status and vitamin A was found to be statistically significant (*P* < 0.001).

## Discussion

India was one of the first countries in the world to have launched a vitamin A supplementation (VAS) program. In spite of this leadership role of the country in initiating the program, vitamin A-first dose supplement coverage was found to be low (37.6%) in this study. When vitamin A-first dose supplement was analyzed along with other selected variables a significant association was found amongst Hindu child, born in health institution, and with birth order one, suggestive of higher level of awareness, motivation, and/or better socioeconomic status.

A review of literature corroborated the observation of low vitamin A supplementation coverage. Rapid Household Survey (RHS) reported similar results with coverage at 35%.[8] According to the National Family Health Survey (NFHS-3), Delhi recorded a low coverage of 17.1% of the children having received vitamin A dose in the last six months (2005–06). Taneja reported that only 37.8% children in Delhi had received vitamin A first-dose supplement.[9] Annual report of ministry of health and family welfare, GoI (2005–06) also mentioned the coverage of vitamin A first dose as 44%. It is noted that over the years no improvement in vitamin A coverage in Delhi has been observed. Further, it was noted that even though 30 children were completely immunized for vaccine-preventable disease up to the age of one, they had not received vitamin A supplement, suggestive of missed opportunity. This further corroborates the fact that “access” to health system does not necessarily translate into delivery of quality services to beneficiaries.

A large proportion of the Indian population receives less than 50% of the recommended dietary intake of vitamin A from dietary sources.[10] In the absence of improved dietary intake or fortification strategy, it is clear that vitamin A supplementation is a necessary intervention to compensate for the shortfall in recommended dietary allowance, especially for the community residing in slums. To conclude, the study reflects low vitamin A-first dose coverage in children residing in the slums of Delhi and requires appropriate corrective measures.

## Annexure-I

**Table:** Slum clusters in south municipal zone, Delhi

Name/address of slum clusters		Pop.	Cum. Pop.
1	Indira Gandhi Camp, Block A, Begampur	3000	3000
2	Indira Gandhi Camp, Block B, Begampur	4416	7416
3	Lai Gumbad Camp, Malviya Nagar	2000	9416
4	Valmiki Camp, Begampur	1200	10616
5	Harizan Camp, Begumpur	2500	*13116
6	Jugdamba Camp, Block-A, Malviya Nagar	2556	15672
7	Jugdamba Camp, Block-B, Malviya Nagar	1444	17116
8	Soami Nagar, Jhuggis	1015	18131
9	Guluk wall Maszid, Jhuggis	1207	19338
10	Jamrud Pur, Jhuggis	3555	22893
11	Madrasi Camp, L Block, Kailash Colony	650	23543
12	JJ Cluster Mohammadpur	2000	*25543
13	JJ cluster Arjun Nagar	4500	30043
14	Dr. Ambedkar Basil, Block-A, Sect-l, RK Puram	2250	32293
15	Dr. Ambedkar Basil, Block-B, Sect-l, RK Puram	3352	35645
16	Dr. Ambedkar Basil, Block-C, Sect-l, RK Puram	5602	*41247
17	J.D Leprosy colony, sect-1, RK Puram	215	41462
18	Hanuman Camp, Sector-l, R.K. Puram	1500	42962
19	Ravldass camp, sector-1 , RK Puram	1200	44162
20	Nepali Camp, sect-1 , RK Puram	560	44722
21	Ekta Vihar, Sector-VI, R.K.Puram	3000	47722
22	JPCol. Nehru Ekta Camp, Sect-VI, R.K.Puram	2500	50222
23	J.J. Colony, Malai Mandir, Sect-VII, R.K.Puram	1000	51222
24	Sonia Camp, Sector-VII, R.K.Puram.	1000	52222
25	K.D. Colony, Block-1,Sector-XII, R.K. Puram	3359	*55581
26	K.D. Colony, Block-2,Sector- XII, R.K. Puram	1547	57128
27	K.D. Colony, Block-3,Sector- XII, R.K. Puram	1210	58338
28	K.D. Colony, Block-4,Sector- XII, R.K. Puram	4100	62438
29	Shastri Market. JJ Cluster, Block A, Nanak Pura	3000	65438
30	Sashtri Market. JJ Cluster, Block B, Nanak Pura	2500	67938
31	Bhanvarsingh camp, Vasant vihar	1500	*69438
32	Khanpur Extension	2500	71938
33	J.J. Colony, Block A, Khanpur	2500	74438
34	J.J. Colony, Block B, Khanpur	1400	75838
35	J.J. Colony, Block C, Khanpur	1500	77338
36	J.J. Colony, Block D, Khanpur	2000	79338
37	Ambedkar Colony	2500	81838
38	Nutt colony, Chhattapur Extension	2700	*84538
39	Harsarup Colony	2500	87038
40	Bhlm Bastl, Jaunapur	4000	91038
41	Shantl Camp, Mandl Gaon	3000	94038
42	Bapu camp, Mandl Gaon	2100	96138
43	Shambu camp Mandi Gaon	3250	*99388
44	Aya Nagar Extension	3500	102888
45	Janta Jiwan Camp, Block A, Devil Road, Tigri	2500	105388
46	Janta Jiwan Camp, Block A-1 , Devil Road, Tigri	2250	107638
47	Janta Jiwan Camp, Block B, Devil Road, Tigri	2230	109868
48	Janta Jiwan Camp, Block B-1 , Devil Road, Tigri	2563	*112431
49	Janta Jiwan Camp, Block C Devil Road, Tigri	2541	114972
50	Janta Jiwan Camp, Block C-1 , Devli Road, Tigri	2252	117224
51	Janta Jiwan Camp, Block D, Devli Road, Tigri	2290	119514
52	Janta Jiwan Camp, Block D-1 , Devli Road, Tigri	3000	122514
53	Janta Jiwan Camp, Block E, Devli Road, Tigri	3500	*126014
54	Janta Jiwan Camp, Block E-1, Devli Road, Tigri	2500	128514
55	Janta Jiwan Camp, Block-F, Devli Road, Tigri	2460	130974
56	Janta Jiwan Camp, Block-F-1 , Devli Road, Tigri	1453	132427
57	Janta Jiwan Camp, Block-G, Devli Road, Tigri	1211	133638
58	Janta Jiwan Camp, Block G-1 , Devli Road, Tigri	1200	134838
59	Janta Jiwan Camp, Block-H, Devli Road, Tigri	1200	136038
60	J.J. Colony, Block-1 , Tigri	2800	138838
61	J.J. Colony, Block-2, Tigri	2565	*141403
62	J.J. Colony, Block-3, Tigri	2400	143803
63	J.J. Colony, Block-4, Tigri	2100	145903
64	J.J. Colony, Block-5, Tigri	2366	148269
65	J.J. Colony, Block-6, Tigri	2300	150569
66	J.J. Colony, Block-7, Tigri	2890	153459
67	J.J. Colony, Block-8, Tigri	3100	*156559
68	J.J. Colony, Block-9, Tigri	3111	159670
69	J.J. Colony, Block-10, Tigri	3500	163170
70	J.J. Colony, Tigri Extension	2800	165970
71	JJ colony, Block A, Sangam Vihar	6500	*172470
72	JJ colony, Block B, Sangam Vihari	7000	179470
73	JJ colony, Block C, Sangam Vihar	6257	*185727
74	JJ colony, Block D, Sangam Vihar	6600	192327
75	JJ colony, Block E, Sangam Vihar	7566	*199893
76	JJ colony, Block F, Sangam Vihar	7410	207303
77	JJ colony, Block G, Sangam Vihar	6500	*213803
78	JJ colony, Block H, Sangam Vihar	6660	220463
79	JJ colony, Block I, Sangam Vihar	7565	*228028
80	JJ colony, Block J, Sangam Vihar	7555	235583
81	JJ colony, Block K, Sangam Vihar	8200	*243783
82	JJ colony, Block L, Sangam Vihar	7775	251558
83	JJ colony, Block M, Sangam Vihar	7532	*259090
84	JJ colony, Block A, Dakshin Puri	6880	265970
85	JJ colony, Block B, Dakshin Puri	6880	*272850
86	JJ colony, Block C, Dakshin Puri	6333	279183
87	JJ colony, Block D, Dakshin Puri	7112	*286295
88	JJ colony, Block E, Dakshin Puri	7533	293828
89	JJ colony, Block F, Dakshin Puri	7755	*301583
90	JJ colony, Block G, Dakshin Puri	7850	309433
91	JJ colony, Block H, Dakshin Puri	7880	*317313
92	JJ colony, Block I, Dakshin Puri	7230	324543
93	JJ colony, Block J, Dakshin Puri	7521	*332064
94	JJ colony, Block K, Dakshin Puri	6711	338775
95	Harijan camp Khanpur	1236	*340011
96	Shri Ram Camp, South Camp	3100	343111
97	Moti Lai Nehru Camp, J.N.U	3560	346671
98	Indira Camp, Bhatti Mines	2850	349521
99	Balbir Nagar, Bhatti Mines	3400	352921
100	Sanjay Camp-1 , Bhatti Mines	3190	*356111
101	Sanjay Camp-2, Bhatti Mines	1750	357861
102	Sanjay Camp-3, Bhatti Mines	4002	361863
103	Sanjay Camp-4, Bhatti Mines	4934	366797
104	Sanjay Camp-5, Bhatti Mines	1100	367897
105	Shaheed camp, Dakshin Puri	1047	*368944
106	Sanjay camp, Dakshin Puri	3963	372907
107	Subhash camp, Dakshin Puri	5740	378647
108	Dalit camp, G block, Dakshin Puri	300	378947
109	Banjara camp, Dakshin Puri	850	379797
110	Harijan camp, lal building, Dakshin Puri	1120	380917
111	Kalyan Samiti Camp, Dakshin Puri	630	381547
112	J.J. Colony, Block-1 , Dakshin Puri	2000	*383547
113	J.J. Colony, Block-2, Dakshin Puri	2500	386047
114	J.J. Colony, Block-3, Dakshin Puri	2530	388577
115	J.J. Colony, Block-4, Dakshin Puri	2360	390937
116	J.J. Colony, Block-5, Dakshin Puri	2133	393070
117	J.J. Colony, Block-6, Dakshin Puri	2500	395570
118	J.J. Colony, Block-7, Dakshin Puri	2140	*397710
119	J.J. Colony, Block-8, Dakshin Puri	2400	400110
120	J.J. Colony, Block-9, Dakshin Puri	2200	402310
121	J.J. Colony, Block-10, Dakshin Puri	2230	404540
122	J.J. Colony, Block-11, Dakshin Puri	2500	407040
123	J.J. Colony, Block-12, Dakshin Puri	2340	409380
124	J.J. Colony, Block-13, Dakshin Puri	2511	411891
125	J.J. Colony, Block-14, Dakshin Puri	2496	*414387
126	J.J. Colony, Block-15, Dakshin Puri	2477	416864
127	J.J. Colony, Block-16, Dakshin Puri	2500	419364
128	J.J. Colony, Block-17, Dakshin Puri	2541	421905
129	J.J. Colony, Block-18, Dakshin Puri	2311	424216
130	J.J. Colony, Block-19, Dakshin Puri	2403	*426619
131	J.J. Colony, Block-20, Dakshin Puri	2511	429130
Sampling interval		429130/30=14304	
Random no.		10866		

Selected slum clusters (N= 30)

## Annexure-II

**Name of the Institute**……………………………[*National Institute of Health and Family Welfare, New Delhi*]

- D.O.B (eligible child):    Household no:
- Religion [Hindu/Non-Hindu]
- Literacy status of mother [Literate/Illiterate]:
- Place of birth [Health Institution/home]:
- Sex [M / F]:
- Birth order:
- Child taken for immunization [Yes/No]:
- Immunization status up to age-one [√]:
  - Complete:
  - Partial:
  - Non-immunized:
- Vitamin A-first dose supplement received [Yes/No]: Put [√] where applicable:

**Table:**

BCG	DPT			OPV				Hepatitis B				Measles	Vitamin A-I
													

	1	2	3	0	1	2	3	0	1	2	3		
